# Characterisation of *PALB2* tumours through whole-exome and whole-transcriptomic analyses

**DOI:** 10.1038/s41523-021-00254-4

**Published:** 2021-04-23

**Authors:** Pei Sze Ng, Jia Wern Pan, Muhammad Mamduh Ahmad Zabidi, Pathmanathan Rajadurai, Cheng Har Yip, Oscar M. Reuda, Alison M. Dunning, Antonis C. Antoniou, Douglas F. Easton, Carlos Caldas, Suet-Feung Chin, Soo Hwang Teo

**Affiliations:** 1 Cancer Research Malaysia, Subang Jaya, Malaysia; 2 University Malaya Cancer Research Institute, Faculty of Medicine, University Malaya, Kuala Lumpur, Malaysia; 3 Subang Jaya Medical Centre, Subang Jaya, Malaysia; 4 Cancer Research UK, Cambridge Institute & Department of Oncology, Li Ka Shing Centre, Robinson Way, Cambridge, UK; 5 Centre for Cancer Genetic Epidemiology, Department of Oncology, University of Cambridge, Cambridge, UK; 6 Centre for Cancer Genetic Epidemiology, Department of Public Health and Primary Care, University of Cambridge, Cambridge, UK; 7 Cambridge Breast Cancer Research Unit, CRUK Cambridge Cancer Centre, Cambridge, UK; 8 NIHR Cambridge Biomedical Research Centre and Cambridge Experimental Cancer Medicine Centre, Cambridge University Hospital NHS Foundation Trust, Cambridge, UK

**Keywords:** Breast cancer, Cancer genomics

## Abstract

Rare protein-truncating variants (PTVs) in *PALB2* confer increased risk to breast cancer, but relatively few studies have reported the characteristics of tumours with *PALB2* PTVs. In this study, we describe molecular characteristics of tumours with either germline or somatic alterations in *PALB2*. DNA from fresh frozen tumour tissues and matched peripheral blood lymphocytes for 560 breast cancer patients was subjected for whole-exome sequencing (WES), and RNA from tumour tissues was subjected to RNA sequencing (RNA-seq). We found six cases with germline and three with somatic protein-truncating variants in *PALB2*. The characteristics of tumours in patients with *PALB2* PTVs were similar to those with *BRCA1* and *BRCA2* PTVs, having significantly more somatic alterations, and a high proportion of the mutational signature and genomic scar scores characteristic of deficiencies in homologous recombination (HR), compared to tumours arising in non-carriers. Unlike tumours arising in patients with *BRCA1* and *BRCA2* PTVs, *PALB2* tumours did not have high prevalence of *TP53* somatic alterations or an enriched immune microenvironment. In summary, *PALB2* tumours show the homologous recombination deficiencies characteristic of *BRCA1* and *BRCA2* tumours, and highlight the potential clinical relevance of *PALB2* mutational status in guiding therapeutic choices.

## Introduction

PALB2 [Partner and Localizer of BRCA2] plays a vital role in the maintenance of genome integrity and repair of DNA double-strand breaks via homologous recombination (HR) pathway, by localising BRCA2 to the sites of DNA damage and serving as a linker between BRCA1 and BRCA2^1,2^. Biallelic (homozygous) germline protein-truncating variants (PTVs) in *PALB2* result in Fanconi anaemia^3^, whereas monoallelic (heterozygous) PTVs predispose individuals to breast, ovarian and pancreatic cancers^4,5^.

In addition to the use of germline testing for *PALB2* for management of risk to breast and other cancers, there is increasing interest in exploring the potential impact of *PALB2* variants on response to PARP inhibitors. Two recent studies using formalin-fixed tissues show that a significant proportion of the tumours arising in *PALB2* loss of function germline carriers have a loss of the second allele and biallelic loss of *PALB2* results in the acquisition of genomic characteristics consistent with deficiency in double-strand DNA break repair^6,7^. However, in part because of the rarity of germline carriers, there has hitherto been no reports of genomic analyses from fresh frozen tumour samples.

In this study, we report the genomic and transcriptomic characteristics of fresh frozen and formalin-fixed paraffin-embedded tumours with *PALB2* alterations, in comparison to the tumours with *BRCA1* or *BRCA2* alterations and non-carriers.

## Results

### Characteristics of tumours arising in *PALB2* carriers

**Fig. 1:**
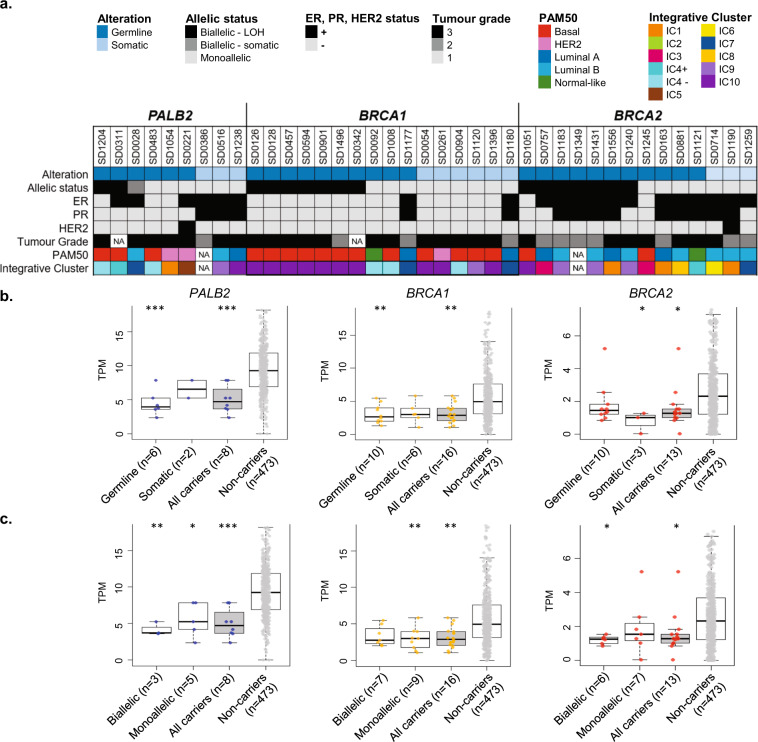
Characteristics of tumours with germline and somatic alterations in *PALB2, BRCA1* and *BRCA2*. **a** Phenobar showing the allelic status, pathology and molecular characteristics of tumours. **b** Comparison of gene expression between tumours with germline and somatic PTV alterations, and tumour with no alterations (non-carriers). **c** Comparison of gene expression between tumours with biallelic inactivation, monoallelic inactivation and tumour with no alterations (non-carriers). Boxplots represent medians (centre line) and interquartile range, and whiskers represent the maximum and minimum values within 1.5 times the interquartile range from the edge of the box. Each data point represents an individual sample. *P* values: Comparison of each category vs non-carrier. ***p < 0.001; **p < 0.01; *p < 0.05; Mann–Whitney *U* test.

Of the 560 breast tumours with available sequencing data, subsequent genomic and transcriptomic profiling was conducted only for samples that passed quality checks [WES, *n* = 546; sWGS, *n* = 533; RNA-seq, *n* = 527]. Germline sequencing identified six individuals with *PALB2* PTVs, 10 with *BRCA1* PTVs and 11 with *BRCA2* PTVs. Whole-exome sequencing (WES) data of tumour DNA identified somatic PTVs in *PALB2* in a further 3 tumours, *BRCA1* in six tumours and *BRCA2* in three tumours (Fig. 1a and Supplementary Table 1). One tumour from an individual with a germline *PALB2* PTV also had a somatic *PALB2* PTV (likely biallelic inactivation via somatic inactivation of the second allele). For all subsequent analyses, tumours with germline or somatic PTVs were considered together. Tumours with germline and somatic missense variants in *PALB2* (*n* = 7), *BRCA1* (*n* = 2) and *BRCA2* (*n* = 11) were excluded.

Of the nine tumours with germline or somatic *PALB2* PTVs, three were triple negative, one was ER-positive/PR-negative/HER2-positive, three were ER-positive/PR-positive/HER2-negative, and two were ER-positive/PR-negative/HER2 negative. For the tumours where data were available (*n* = 8), all the tumours were either grade 2 or 3 (Fig. 1a). Tumours with PTVs in *BRCA1* were more likely to be negative for the oestrogen receptor and progesterone receptor by IHC (14/16 both ER- and PR-), and those with *BRCA2* PTV were more likely to be positive for these receptors (12/14 ER+, 9/14 PR+). Using RNA-seq analysis, we did not observe any enrichment of any subtype in tumours with *PALB2* germline or somatic alterations. This is in contrast with *BRCA1* tumours which were strongly enriched for ‘basal’ and ‘IntClust 10’ subtype^8^, and *BRCA2* tumours which were slightly enriched for Luminal B subtype (Fig. 1a and Supplementary Fig. 1).

Three (33%) of 9 *PALB2* tumours had either loss of *PALB2* wild-type allele (two tumours) or somatic inactivation of the second allele (one tumour) (Fig. 1a and Supplementary Table 2). This rate was lower when compared with 44% of tumours with biallelic inactivation of *BRCA1* (7/16) and 50% of tumours with biallelic inactivation of *BRCA2* (7/14) (Fig. 1, Supplementary Tables 1 and 2). Overall, tumours with germline alterations in *PALB2* and *BRCA1* had significantly reduced gene expression of the respective genes when compared to tumours without alterations, whereas there was no statistically significant difference in expression of these genes in tumours with somatic mutations. In contrast, *BRCA2* tumours with somatic mutations had a significantly lower gene expression levels compared to expression of tumours arising in non-carriers (Fig. 1b). Tumours with biallelic inactivation of *PALB2* and *BRCA2*, and monoallelic *BRCA1* tumours had a significantly lower expression of the respective genes when compared to tumours with no alterations (non-carriers) (Fig. 1c).

**Fig. 2:**
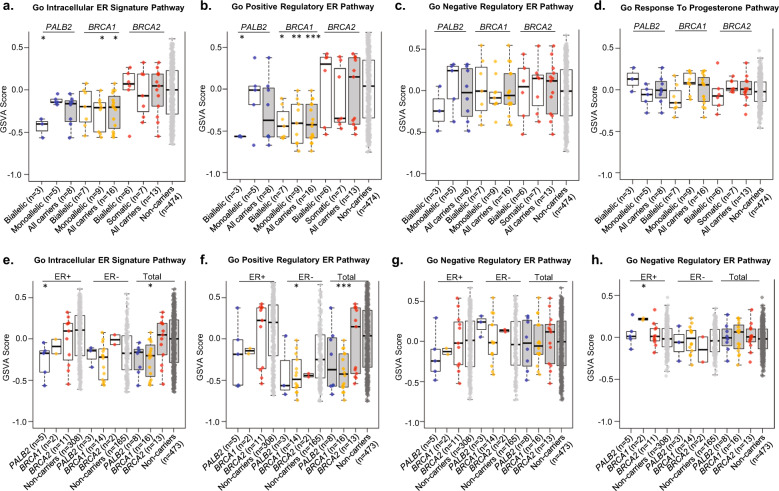
ER and PR pathway analysis, stratified by allelic status (**a**–**d**) and hormone status (**e**–**h**). **a** GO intracellular ER signature pathway. **b** GO positive regulatory ER pathway. **c** GO negative regulatory ER pathway. **d** GO response to progesterone pathway (only samples with available RNA-seq data were included in this analysis). **e** GO intracellular ER signature pathway. **f** GO positive regulatory ER pathway. **g** GO negative regulatory ER pathway. **h** GO response to progesterone pathway. Boxplots represent medians (centre line) and interquartile range, and whiskers represent the maximum and minimum values within 1.5 times the interquartile range from the edge of the box. Each data point represents an individual sample. *P* values: Comparison of each category vs non-carrier. ***p < 0.001; **p < 0.01; *p < 0.05; Mann–Whitney *U* test (bootstrap analysis was applied).

We compared the expression of genes in the ER pathway for *PALB2* tumours with that of *BRCA1* and *BRCA2* tumours. Tumours with biallelic and monoallelic inactivation of *BRCA1* had lower expression of genes regulated by the estrogen receptor (Fig. 2a), and this was particularly striking for genes that are positively regulated by the estrogen receptor (Fig. 2b). Tumours with biallelic, but not monoallelic inactivation of *PALB2*, had similar lower expression of the genes regulated by the estrogen receptor (Fig. 2a, b). By contrast, there was no difference in expression of these genes in tumours with either biallelic or monoallelic inactivation of *BRCA2*, nor in genes negatively regulated by the estrogen receptor (Fig. 2c) or in the progesterone pathway (Fig. 2d).

Given that *BRCA1* tumours are more likely to be ER- negative, we examined the expression of genes regulated by the estrogen receptor pathway by ER subtype. Although the sample size was small, this exploratory analysis showed that the expression of genes positively regulated by estrogen receptor was numerically lower in *PALB2* and *BRCA1* tumours in both ER-positive and ER-negative disease (Fig. 2f), but there was no difference in expression of genes negatively regulated by estrogen receptor (Fig. 2g) or in the progesterone pathway (Fig. 2h).

Next, we conducted an exploratory analysis to determine the key differentially expressed genes in *PALB2, BRCA1* and *BRCA2* tumours compared to tumour in non-carriers. We found 709, 3297 and 1760 genes that were significantly differentially expressed between *PALB2*, *BRCA1* and *BRCA2* tumours compared to non-carriers. Intriguingly, a large proportion of the differentially expressed genes identified in *PALB2* tumours (98.2%, 696/709) overlapped with the differentially expressed genes identified in *BRCA1* and *BRCA2* tumours. Gene Ontology (GO) functional enrichment analysis demonstrated that the upregulated differentially expressed genes for *PALB2* tumours were enriched in the molecular function and cellular component terms associated with RNA and protein binding. Pathway enrichment analysis showed that the top results were enriched in metabolism of RNA, transcription, translation and metabolism of protein which was highly similar with what were observed in *BRCA1* and *BRCA2* tumours (Supplementary Fig. 2).

### Mutational profiles of *PALB2* tumours

**Fig. 3:**
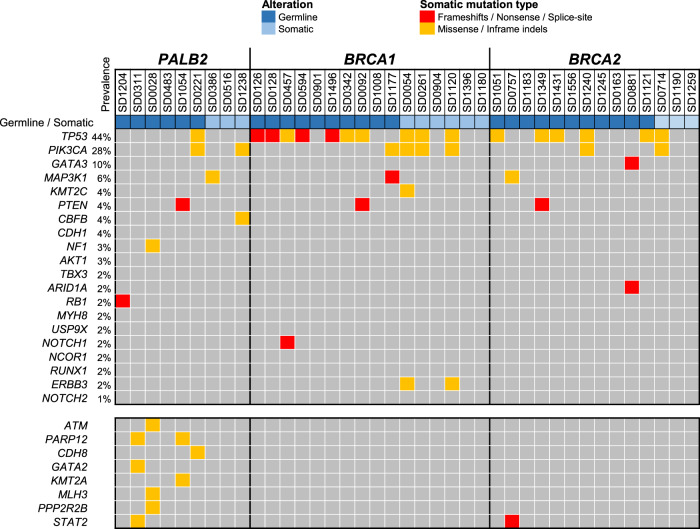
The oncoplot shows all the somatic mutations identified in *PALB2* tumours in comparison to *BRCA1* and *BRCA2* tumours in the top 20 mutated genes commonly associated with breast cancer (with prevalence indicated as reported previously by ^18^). The lower bottom panel represented somatic alterations in *PALB2* tumours identified in other additional genes. Each column represented a sample.

Using WES analyses, we characterised the prevalence of driver gene mutations in tumours with germline and somatic alterations in *PALB2*, *BRCA1* and *BRCA2*. *TP53* somatic mutations were found in 11% of *PALB2* tumours (1/9), compared to 63% of *BRCA1* tumours (10/16), and 43% of *BRCA2* tumours (6/14) (Fig. 3). *PIK3CA* somatic mutations were found in 22% of *PALB2* tumours (2/9), compared to 25% of *BRCA1* tumours (4/16), and 14% of *BRCA2* tumours (2/14). There were no other commonly mutated driver genes found in *PALB2* tumours (Fig. 3).

**Fig. 4:**
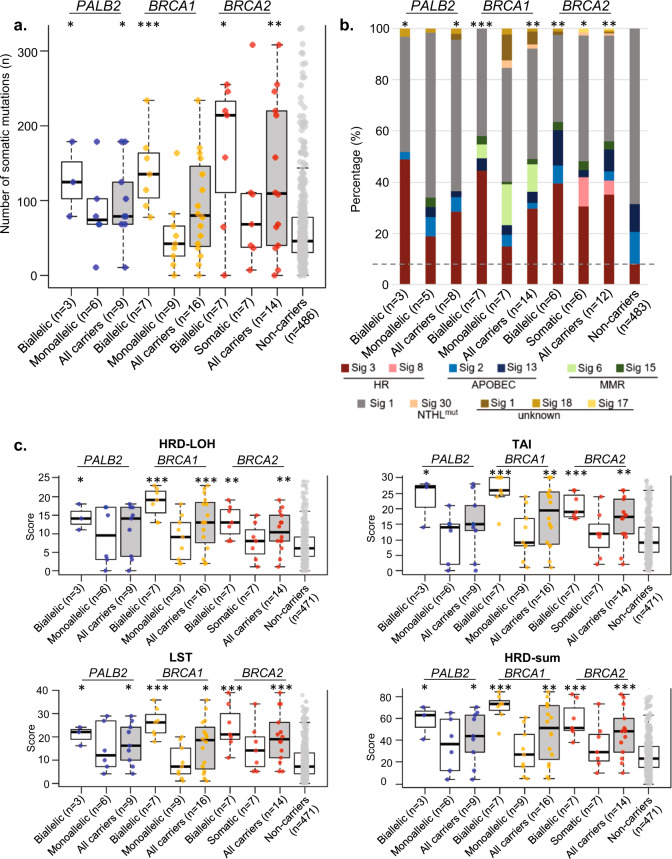
Comparison of mutational profiles of tumours (with integrated germline and somatic alterations) that arise from mutation carriers of *PALB2, BRCA1* and *BRCA2* and non-carriers, stratified by allelic status of tumours. **a** Total number of somatic alterations (single nucleotide variants (SNVs)) and indels identified in tumours with PTVs. **b** The stacked bar plot shows the proportion of major mutational signature in aggregate for each category. The horizontal grey dashed line indicates the proportion of mutational signature 3 in tumours that arise from non-carriers as reference for comparison. Only samples with at least 15 SNVs were included in this analysis. **c** Comparison of genomic scar scores for tumours with PTVs and tumours without any alterations (non-carrier group). All samples were included in this analysis except those with no Sequenza files due to unavailability of either WES germline or tumour data. Boxplots represent medians (centre line) and interquartile range, and whiskers represent the maximum and minimum values within 1.5 times the interquartile range from the edge of the box. Each data point represents an individual sample. *P* values: Comparison of each category vs non-carrier. ***p < 0.001; **p < 0.01; *p < 0.05; Mann–Whitney *U* test (**a**, **c**); Chi-square test (**b**).

Using WES data, we determined the total number of somatic mutations [small insertion–deletions (indels) and single nucleotide variations (SNVs)] for each tumour sample. Tumours with biallelic inactivation of *PALB2*, *BRCA1* and *BRCA2* had a significantly higher number of somatic mutations compared to tumours of non-carriers (median 125, 146 and 214 respectively compared to 46 in non-carriers, *p* = 0.0364, 0.0009 and 0.0137, respectively). However, the number of somatic mutations was not significantly different in tumours with monoallelic inactivation of *PALB2*, *BRCA1* or *BRCA2* compared to tumours of non-carriers [median 74, 42 and 68 respectively compared to 46 in non-carriers, *p* = 0.2026, 0.5362 and 0.4258, respectively, Fig. 4a].

Next, we determined the proportion of the major mutational signatures in the tumour samples (Supplementary Figure 3). Tumours with biallelic inactivation of *PALB2, BRCA1* or *BRCA2* had a higher proportion of mutational signature 3 [mean 48.9%, 44.4% and 39.5% respectively compared to 8.2% in non-carriers, *p* = 0.0114, 0.0007 and 0.0064, respectively; Fig. 4b]. However, the proportion of mutational signature 3 was not significantly different in tumours with monoallelic inactivation of *PALB2*, or *BRCA1* compared to tumours of non-carriers [mean 18.8% and 15.1% respectively compared to 8.2% in non-carriers, *p* = 0.3881 and 0.5092, respectively, Fig. 4b], whereas that in tumours with monoallelic inactivation of *BRCA2* was marginally higher than that in non-carriers [mean of 30.6% compared to 8.2% in non-carriers, *p* = 0.0491].

We examined the other features of HR deficiency including genomic loss of heterozygosity (LOH), telomeric allelic imbalance (TAI) and large-scale state transition (LST). Tumours with biallelic inactivation of *PALB2*, *BRCA1* and *BRCA2* had a significantly higher scores of LOH compared to tumours of non-carriers (median 14, 19 and 13 respectively compared to 6 in non-carriers, *p* = 0.0171, 0.0002 and 0.0015, respectively). Tumours with biallelic inactivation of *PALB2*, *BRCA1* and *BRCA2* also had a significantly higher TAI scores compared to tumours of non-carriers (median 27, 26 and 19 respectively compared to 9 in non-carriers, *p* = 0.0164, 0.0002 and 0.0002 respectively). Tumours with biallelic inactivation of *PALB2*, *BRCA1* and *BRCA2* had a significantly higher large-scale transition scores compared to tumours of non-carriers (median 22, 26 and 21 respectively compared to 7 in non-carriers, *p* = 0.0200, 0.0002 and 0.0003, respectively). Overall, the HRD scores (HRD-sum) was higher for tumours with biallelic inactivation of *PALB2*, *BRCA1* and *BRCA2* compared to non-carriers. However, none of these measures were significantly different in tumours with monoallelic inactivation of *PALB2*, *BRCA1* or *BRCA2* compared to tumours of non-carriers (Fig. 4c).

### Immune profiles of *PALB2* tumours

**Fig. 5:**
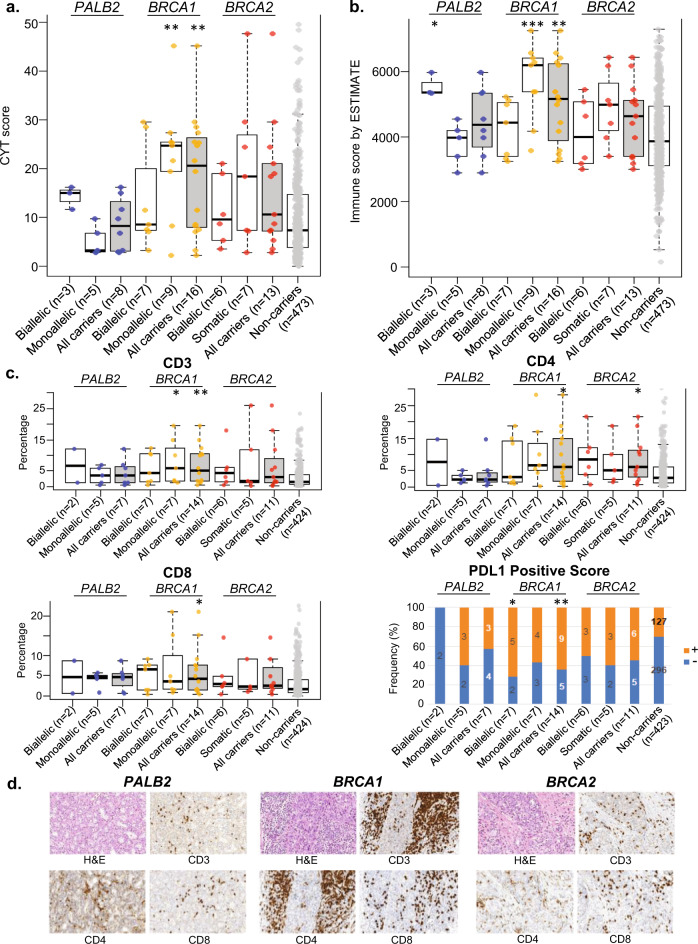
Comparison of immune profiles of tumours (with integrated germline and somatic alterations) that arise from mutation carriers of *PALB2*, *BRCA1* and *BRCA2* and non-carriers. **a** Cytolytic index (CYT), as measure of CD8+ T cell cytotoxicity. **b** ESTIMATE score, as measure of lymphocytic infiltration. **c** Distribution of TILs and PD-L1 expression. Only samples with IHC images for analysis were included in this analysis. **d** Representative images of H&E and IHC for CD3, CD4 and CD8 where image analysis was performed. Boxplots represent medians (centre line) and interquartile range, and whiskers represent the maximum and minimum values within 1.5 times the interquartile range from the edge of the box. Each data point represents an individual sample. *P* values: Comparison of each category vs non-carrier. **p < 0.01; *p < 0.05; Mann–Whitney *U* test (CD3, CD4 and CD8); Chi-square test (PD-L1 positive score).

We examined the immune tumour microenvironment through bioinformatics analysis of immune-related genes, and found that tumours with monoallelic inactivation of *BRCA1* had higher CD8 positive T-cell cytotoxicity (as measured by cytolytic (CYT) index; Fig. 5a) and a higher immune infiltrate (as measured by ESTIMATE score; Fig. 5b). Tumours with biallelic inactivation of *PALB2* had higher immune infiltrate (Fig. 5b), but all other tumours did not have increased immune profiles (Fig. 5a, b).

We retrieved and performed immunohistochemistry (IHC) on the corresponding formalin-fixed paraffin-embedded tumour samples and found that *BRCA1* tumours had higher levels of CD3, CD4, CD8 and PD-L1 positive staining compared to non-carriers, but there were no significant differences in the percentage of TILs or PD-L1 expression of *BRCA2* and *PALB2* tumours compared to non-carriers (Fig. 5c).

### Survival analyses

Given the association between mutation status and immune profiles, we explored the association between mutation status and breast cancer survival. We found that women with *PALB2* PTVs had poorer survival compared to *BRCA* carriers and non-carriers, but the result was not statistically significant (Supplementary Fig. 4).

## Discussion

This study describes the characteristics of tumours that arise from *PALB2* carriers based on the integration of genomics and transcriptomics analysis in fresh-frozen tumour samples. Despite the relatively small sample size, our analyses show that tumours with biallelic inactivation in *PALB2* are similar to that with biallelic inactivation in *BRCA1* or *BRCA2* in that they are of high grade, had higher mutational load^9,10^ and appear to display mutational signatures^9–13^ and chromosomal instabilities that are characteristic of loss of the HR pathway^14,15^. Intriguingly, we found that tumours with biallelic inactivation in *PALB2* and *BRCA1*, appear to have downregulation of genes regulated in the estrogen receptor pathway and that the majority of the differentially expressed genes are similarly dysregulated in *BRCA1* and *BRCA2* tumours. However, unlike *BRCA1* tumours, *PALB2* tumours do not appear to display an enriched immune microenvironment. Taken together, these data suggest that biallelic loss of *PALB2* may result in tumour features that are broadly similar to that of biallelic loss of *BRCA1*, adding further weight to the body of evidence that carriers of *PALB2* alterations should be considered for therapies, which have been approved for use in germline carriers of *BRCA1* and *BRCA2*, such as PARP inhibitors^14^.

Our results are consistent with previous studies in tumours with biallelic inactivation of *PALB2* using targeted sequencing or whole-exome analysis of paraffin-embedded tissue. Notably, the use of WES has enabled us to characterise genome-wide mutational load, which adds to the previous analyses involving targeted sequencing of ~500 genes^7^. In addition, the analysis of tumours with *PALB2*, *BRCA1* and *BRCA2* alterations collected at the same centre and analysed at the same time with the same platform, confirms previous WES analyses conducted on paraffin-embedded material where the TCGA dataset analysed from frozen material on a different platform was used as a comparator^6^. Taken together, the results show that biallelic inactivation of *PALB2* is associated with the higher mutation load, the higher proportion of mutational signature 3, or the higher genomic scar scores indicative of genomic instability.

However, there are some differences between our results and previous published studies. The prevalence of biallelic loss of *PALB2* at 50% of germline carriers is slightly lower than the 67% reported in previous studies^6,7^. Whilst this may be due to chance because of the small dataset, it warrants further investigation as the tumour features appear to be associated with biallelic loss of *PALB2* and is not statistically significant in tumours with monoallelic loss of *PALB2*. In addition, the frequency of somatic *TP53* mutations in *PALB2* tumours (1/9, 11%) was lower than that reported previously using deeper targeted sequencing approaches [21% (5/24) and 40% (6/15)] of *PALB2* tumours, respectively^6,7^. However, the prevalence of *PIK3CA* somatic mutations in *PALB2* tumours (2/9, 22%) was consistent with that previously reported (29%)^6^.

Finally, this study explored the immune microenvironment in *PALB2* tumours. Whilst we found an enrichment in immune genes, as determined using the ESTIMATE immune profiles, this was not found when we examined CYT scores, nor immunohistochemical staining with CD3, CD4, CD8 or PD-L1. These results suggest that there may be unique characteristics of *BRCA1*-associated tumour that modulates their enriched immune microenvironment, that is distinct from the shared functions that *BRCA1* shares with *PALB2* and *BRCA2* in genomic instability^16^.

This study has several limitations. Despite being a unique study with fresh frozen tumour genomic analyses, the number of samples available for analyses was small. Hence some differences in tumour characteristics between *PALB2* carriers and non-carriers may have been missed, and the frequencies of specific characteristics could only be crudely estimated. In particular, the lower frequency of immune infiltration and relatively high frequency of *PIK3CA* mutations need to be confirmed in larger studies. Further studies using larger sample sizes should define the distribution of genomic features more precisely, and hence provide better understanding of the role of *PALB2* germline and somatic variants in the oncogenesis.

In conclusion, this study has demonstrated similarities and highlighted potential differences between tumours arising in *PALB2* PTV carriers with that of *BRCA1* and *BRCA2* carriers. In particular, these results confirm that biallelic loss of *PALB2* results in tumour characteristics, which may be sensitive to therapies targeting the HR pathway in ways that are similar to biallelic loss of *BRCA1* and *BRCA2*.

## Methods

### Study subjects and biospecimen acquisition

Breast tumour and peripheral blood specimen were obtained from 656 patients diagnosed with breast cancer who underwent surgical resection in Subang Jaya Medical Centre between September 2012 and March 2017. The study participants were women with breast cancer who were recruited in the Malaysian Breast Cancer Genetic (MyBrCa) study^17^. Representative fresh tumour tissues excised from the primary tumour were collected at surgery, immediately frozen and stored in liquid nitrogen. Recruitment and genetic studies have been approved by the Ethics Committee of Subang Jaya Medical Centre [reference no: 201208.1] and written informed consent was given by each participant.

During cryo-sectioning, two 8-µm frozen sections were collected from each tissue (at the beginning and end of sectioning), placed on the same slide (Polysine slides, Thermo Scientific, UK) and thereafter stained with Hematoxylin & Eosin (H&E) stain [Hematoxylin Harris (Product code: 351945 S, BDH, USA); Eosin Y solution (Product code: 1098441000, Merck, Germany)]. Subsequently, ten 30-µm sections were taken in an alternate manner from each tumour, placed into two sets of tubes pre-filled with the respective lysis buffer (supplied with the DNeasy Blood and Tissue Kit and miRNeasy Kit (Qiagen, Crawley, UK)) and kept frozen in −80 °C for nucleic acid extractions at later time. The stained H&E slides were reviewed under a light microscope to determine the tumour content (by taking the average of both sections). Only cases with average tumour content ≥30% and with sufficient quality and quantity of nucleic acid were selected and subjected for sequencing as described previously. Additional cases were excluded from this study for the following reasons: no corresponding germline samples, those that withdrew consent from the study and those with rare histology subtypes that were not suitable to be included in this study (mucinous, malignant phylloides).

### Nucleic acid isolation, quantification, and quality assessment

DNA from blood samples was extracted using the Maxwell 16 Blood DNA Purification Kit with the Maxwell 16 Instrument (Promega, Madison, WI, USA) according to standard protocol. DNA and RNA were extracted and purified from ten 30-µm sections each from fresh frozen tumours using the DNeasy Blood and Tissue Kit and the miRNeasy Kit (Qiagen, Crawley, UK) on the QIAcube (Qiagen) according to the manufacturer’s instructions. The purity and quantity of nucleic acids were quantified with a NanoDrop ND-2000 spectrophotometer (NanoDrop Technologies, Wilmington, DE, USA) and the integrity of random DNA samples was assessed by agarose gel electrophoresis. Purified DNA were further quantified by flourometry using the Qubit dsDNA HS [high sensitivity] Assay kit with Qubit 2.0 fluorometer (Thermo Scientific). RNA integrity was assessed using the Agilent 2100 Bioanalyser Nanochip (Agilent Technologies, Wokingham, UK). For DNA samples, only tumour samples with a concentration above 20 ng/µL were included for WES, whilst for RNA, only samples with concentration of 10 ng/µL with RNA integrity number of 7 and above were included for whole-transcriptomic sequencing, respectively.

### Sequencing of germline and tumour samples

Germline DNA, tumour DNA and RNA were subjected for library preparation and sequenced as described previously^18^. Briefly, for the WES, DNA libraries were generated from 50 ng of genomic DNA using the Nextera Rapid Capture Exome kit and subjected to paired end 75 base pair sequencing on the Hi-Seq 4000 platform (Illumina, San Diego, USA). In addition, 4 nM pools of DNA libraries was subjected to shallow whole-genome sequencing (sWGS). Exome capture was performed in pools of 3 and subjected to paired end 75 sequencing on a HISEQ4000 platform (Illumina, San Diego, USA). For RNA-seq, RNA libraries were prepared from 550 ng of total RNA using the TruSeq Stranded Total RNA HT kit with Ribo-Zero Gold (Illumina, San Diego, USA) and subjected to paired end 75 base pair sequencing on a Hi-Seq 4000 (Illumina, San Diego, USA).

### Bioinformatics analysis

Analysis of sequencing data was performed as described previously^18^. Briefly, for WES, sequenced reads were aligned to the human reference genome GRCh37 using BWA-MEM^19^. Local realignment, duplicate removal and base quality recalibration were performed using the Genome Analysis Toolkit (GATK, v3.1.1)^20^. Somatic SNVs were detected using GATK3 Mutect2^20^, whilst small insertions and deletions (indels) were called by Strelka2^21^. RNA-seq, reads were mapped to the hs37d5 human genome and the ENSEMBLE GrCh37 release 87 human transcriptome using the STAR aligner (v.2.5.3a)^22^. Variant calling for RNA-seq data was also conducted using the GATK Best Practices workflow for RNA-seq.

### Mutational signatures

Only samples with at least 15 SNVs were used to determine the mutational signatures. The weights of previously reported breast cancer mutational signatures from using COSMIC matrices, (Signatures 1, 2, 13, 3, 8, 6, 15, 20, 26, 5, 17, 18 and 30) were determined using deconstructSigs^23^.

### HR deficiency scores

The following measures of HR deficiency were determined as described previously: (1) LOH, (2) LST and, (3) TAI^24–26^. Allele-specific copy number (ASCN) profiles on paired normal-tumour BAM files were determined using Sequenza^27^ and used to calculate the individual measure scores and HRD-sum scores using the scarHRD R package^28^.

### Molecular classification based on gene expression data

Classification into breast cancer subgroups was performed using PAM50^29^ and Integrative Clusters (IntClust)^30^.

### Cytolytic index (CYT) and ESTIMATE score

CYT index (which is a measure of CD8+ T-cell cytotoxicity) was obtained by quantifying the transcript levels of 2 genes*, granzyme A* (*GZMA*) and *perforin 1* (*PRF1*)^31^. ESTIMATE (v. 1.0.13)^32^ gives a measure of immune cell infiltration by performing ssGSEA based on inferred immune signature. The ESTIMATE immune score was obtained by using ‘estimate’ R package.

### Evaluation of tumour infiltrating lymphocytes (TILs) and PD-L1

Formalin-fixed paraffin-embedded (FFPE) blocks for 456 patients with sequencing data were sectioned and stained for anti-CD3 (clone 2GV6, predilute; Ventana Medical Systems), anti-CD4 (clone SD35, predilute; Ventana Medical Systems), anti-CD8 (clone SD57, predilute; Ventana Medical Systems) and anti-PD-L1 (clone SP263, predilute; Ventana Medical Systems) using an automated immunostainer (Ventana BenchMark ULTRA; Ventana Medical Systems, Tucson, AZ). Stained slides were digitised using an Aperio AT2 whole slide scanner. CD3, CD4, and CD8 staining were quantified using the Aperio Positive Pixel digital pathology tool (v9 algorithm at 0.16 colour saturation). PD-L1 expression was determined using the Combined Positive Score system (0: no stain (negative); 1: ≥1% positive tumour cells staining).

### Determination of locus-specific loss of *PALB2*, *BRCA1* and *BRCA2* wild-type allele (locus-specific LOH)

Locus-specific LOH of germline *PALB2*, *BRCA1* and *BRCA2* mutation in the tumour was determined using two methods: 1. ASCN calls^14^ and 2. Allele frequency comparisons^33^. Briefly, ASCN calls of the genomic region containing the *PALB2, BRCA1* and *BRCA2* germline mutation were determined by Sequenza as reported previously^14,27^ and a tumour sample is considered to have LOH if the variant allele frequency in the DNA of the tumour sample was >20% than that in the corresponding germline DNA^33^. In cases where there was difference in both calls, a third method, to determine the genome-wide copy number data using QDNAseq where sWGS data were used as input to substantiate the LOH^34^.

### Differential gene expression and functional enrichment analysis

Gene expression was analysed with the limma package, an R-based open-source software designed to analyse transcriptomic data for differential expression, as previously described^18^. GO enrichment analysis, Kyoto Encyclopaedia of Genes and Genomes pathway enrichment analysis and Reactome pathway analysis were performed using the Database for Annotation, Visualisation and Integration Discovery (DAVID, http://david.abcc.ncifcrf.gov/)^35^. The *p* value was adjusted by Bonferroni correction.

### Statistical analysis

The Mann–Whitney *U* test and the Chi-square test were performed for comparisons of variables between mutation categories. *P* < 0.05 was considered statistically significant and all tests were two-sided. Statistical analyses were performed using R v3.6.1. Bootstrap analysis was performed using R to account for the difference in sample size. In brief, 30 non-carrier controls were randomly selected for comparison with each category of mutation carriers. The process was iterated 1000× and two tailed *p* value was calculated with the Mann–Whitney *U* test for each iteration. The median of all iterations was determined and used as the final corrected *p* value.

### Survival analysis

Overall survival data were obtained for each patient as previously described^18^. The Cox proportional hazard model was built using “coxph’ function from the survival package adjusting for covariates and plotted using the ‘ggforest” function from survminer R package. Adjusted survival curves were calculated based on the Cox model and plotted using “ggadjustedcurves” function.

### Reporting summary

Further information on research design is available in the Nature Research Reporting Summary linked to this article.

## Supplementary information

Supplementary Data 1

Supplementary Information

Reporting Summary

## Data Availability

The data generated and analysed during this study are described in the following data record: 10.6084/m9.figshare.14207165^36^. Sequencing data (WES, RNA-seq and sWGS bam files) are available on the *European Genome-phenome Archive* under the study accession number https://identifiers.org/ega.study:EGAS00001004518^37^. Access to controlled patient data will require the approval of the MyBrCa Tumour Genomics Data Access Committee upon request to the corresponding author at genetics@cancerresearch.my. Characteristics of tumours with germline and somatic PTV identified in *PALB2*, *BRCA1* and *BRCA2* carriers and differentially expressed genes (DEGs) are shared openly as part of the data record^36^.

## Code availability

The R software (version 3.6.1) codes used in the study are available from the corresponding author on request.
