# Supplementary material for: Characterisation of PALB2 tumours through whole-exome and whole-transcriptomic analyses
Source: NPJ Breast Cancer. 2021 Apr 23;7:46. doi: 10.1038/s41523-021-00254-4 (PMC8065101; doi:10.1038/s41523-021-00254-4)
Supplement: Supplementary file 2 — Supplementary Information [file 41523_2021_254_MOESM2_ESM.pdf]

Supplementary information

Supplementary Table 1. Characteristics of tumours with germline and somatic PTVs identified in *PALB2*, *BRCA1* and *BRCA2* carriers.

Supplementary Table 2. List of tumours with germline alteration and status of LOH.

Supplementary Figure 1. Distribution of breast cancer subtype by gene expression data using PAM50 and Integrative Cluster classification.

Supplementary Figure 2. Differentially expressed Genes (DEGs).

Supplementary Figure 3. The stacked bar plot shows the proportion of major mutational signature for each individual mutation carrier.

Supplementary Figure 4. Overall survival in patients with germline and somatic alterations in *PALB2*, *BRCA1* and *BRCA2*.

Supplementary Data File. List of differently expressed genes (DEGs) identified in *PALB2*, *BRCA1* and *BRCA2* tumours.

Supplementary Table 1. Characteristics of tumours with germline and somatic PTVs identified in *PALB2, BRCA1* and *BRCA2* carriers.

Gene	Case ID	Age at diagnosis	Tumour size (cm)	Histology subtype	Alteration type	Variant type	HGVS coding	HGVS protein	Biallelic inactivation	PAM50	IntClust	Availability of IHC for Tils analysis	Tumour content (%)	Variant Allele Freq
PALB2	**SD1204**	71	2.5	IDC	Germline	Frameshift	c.426_428delGCTinsCC	p.(Lys142Asnfs*35)	Yes – LOH	Basal	4-	No	80	0.74
	**SD0311**	41	5.0	ILC	Germline	Nonsense	c.7G>T	p.(Glu3*)	Yes – LOH	Basal	4+	Yes	82.5	0.51
	**SD0028**	64	2.0	IDC	Germline	Frameshift	c.1037_1041delAAGAA	p.(Lys346Thrfs*13)	Yes - Som inactivation	LumB	7	Yes	80	0.50
					Somatic	Frameshift	c.2762delA	p.(Gln921Argfs*2)						0.27
	**SD0483**	54	1.2	IDC	Germline	Frameshift	c.1050_1053delAACA	p.(Thr351Argfs*4)	No	Basal	4-	Yes	40	0.43
	**SD1054**	36	4.0	IDC	Germline	Frameshift	c.2167_2168delAT	p.(Met723Valfs*21)	No	Her2	1	Yes	70	0.72
	**SD0221**	57	3.8	IDC	Germline	Frameshift	c.2760dupA	p.(Gln921Thrfs*7)	No	Her2	5	No	80	0.49
	**SD0386**	65	5	IDC	Somatic	Nonsense	c.1129C>T	p.(Gln377*)	No	NA	NA	Yes	40	0.21
	**SD0516**	60	4.5	IDC	Somatic	Splice site	c.2514+1G>A		No	LumB	9	Yes	62.5	0.35
	**SD1238**	47	3.1	IDC	Somatic	Nonsense	c.1837C>T	p.(Gln613*)	No	LumA	8	Yes	80	0.09
BRCA1	**SD0126**	50	3.8	IDC	Germline	Frameshift	c.686_687delCT	p.(Ser229*)	Yes - LOH	Basal	10	Yes	50	0.80
	**SD0128**	61	9.0	IDC	Germline	Frameshift	c.3869_3870delAA	p.(Lys1290Metfs*4)	Yes - LOH	Basal	10	Yes	85	0.75
	**SD0457**	37	6.0	Medullary	Germline	Nonsense	c.2635G>T	p.(Glu879*)	Yes - LOH	Basal	10	Yes	70	0.89
	**SD0594**	40	4.5	IDC	Germline	Nonsense	c.2635G>T	p.(Glu879*)	Yes - LOH	Basal	10	Yes	45	0.62
	**SD0901**	54	3.0	IDC	Germline	Frameshift	c.3424delG	p.(Ala1142Hisfs*13)	Yes - LOH	Basal	10	Yes	80	0.69
	**SD1496**	30	1.2	IDC	Germline	Frameshift	c.3770_3771delAG	p.(Glu1257Glyfs*9)	Yes - LOH	Basal	10	Yes	85	0.58
	**SD0342**	33	NA	IDC	Germline	Pathogenic MS	c.5072C>A	p.(Thr1691Lys)	Yes - LOH	Basal	10	Yes	35	0.74
	**SD0092**	51	1.5	IDC	Germline	Frameshift	c.3770_3771delAG	p.(Glu1257Glyfs*9)	No	Normal	4-	No	32.5	0.60
	**SD1008**	33	8.0	IDC	Germline	Nonsense	c.4372C>T	p.(Gln1458*)	No	Basal	4-	Yes	45	1.00
	**SD1177**	37	2.5	IDC	Germline	Splice site	c.5406+1_5406+3delGTA	-	No	LumA	7	Yes	30	0.30
	**SD0054**	86	9	IDC	Somatic	Frameshift	c.5503delC	p.(Arg1835Glufs*8)	No	Basal	10	Yes	82.5	0.14
	**SD0261**	42	3.5	IDC	Somatic	Nonsense	c.520C>T	p.(Gln174*)	No	Her2	10	Yes	80	0.24
	**SD0904**	53	2.5	IDC	Somatic	Frameshift	c.3257delT	p.(Leu1086*)	No	Basal	4-	Yes	30	0.14
	**SD1120**	43	3.4	IDC	Somatic	Nonsense	c.5431C>T	p.(Gln1811*)	No	Basal	9	Yes	30	0.22
	**SD1396**	39	0.5	IDC	Somatic	Frameshift	c.3664delG	p.(Glu1222Serfs*13)	No	Basal	10	No	32.5	0.13
	**SD1180**	56	1.5	IDC	Somatic	Frameshift	c.1881_1884dup	p.(Arg629Glnfs*2)	No	LumA	7	Yes	80	0.23
BRCA2	**SD1051**	68	3.7	IDC	Germline	Frameshift	c.4467_4474delAATACTGAinsTGTTTTT	p.(Lys1489AsnfsTer15)	Yes - LOH	Basal	10	Yes	50	0.44
	**SD0757**	72	2.0	IDC	Germline	Splice site	c.-39-1_-39delGA	-	Yes - LOH	LumA	3	Yes	80	0.79
	**SD1183**	47	2.7	IDC	Germline	Frameshift	c.1368_1369delGA	p.(Lys457Alafs*4)	Yes - LOH	LumB	9	Yes	85	0.73
	**SD1349**	36	12.0	IDC	Germline	Frameshift	c.5896dupC	p.(His1966Profs*2)	Yes - LOH	NA	NA	Yes	85	0.83
	**SD1431**	52	3.0	Pleomorphic ILC	Germline	Nonsense	c.7878G>A	p.(Trp2626*)	Yes - LOH	LumB	9	Yes	85	0.77
	**SD1556**	60	2.2	IDC	Germline	Nonsense	c.7878G>A	p.(Trp2626*)	Yes - LOH	LumA	1	Yes	55	0.65
	**SD1240**	54	2.4	IDC	Germline	Frameshift	c.2176delG	p.(Val726Phefs*4)	Yes - LOH	LumB	9	No	60	0.78
	**SD1245**	37	1.5	IDC	Germline	Frameshift	c.1298dupA	p.(Asn433Lysfs*19)	No	Basal	3	Yes	47.5	0.27
	**SD0163**	41	2.5	IDC	Germline	Nonsense	c.2612C>A	p.(Ser871*)	No	LumA	1	Yes	45	0.56
	**SD0881**	33	9.0	IDC	Germline	Nonsense	c.7629T>G	p.(Tyr2543*)	No	LumB	8	Yes	75	0.47
	**SD1121**	45	1.2	IDC	Germline	Frameshift	c.4257delA	p.(Asp1420Ilefs*28)	No	Normal	4+	No	35	0.40
	**SD0714**	49	6.3	IDC	Somatic	Frameshift	c.4808dup	p.(Asn1603Lysfs*6)	No	LumB	6	Yes	80	0.23
	**SD1190**	69	NA	IDC	Somatic	Nonsense	c.1756A>T	p.(Lys586*)	No	LumB	1	No	85	0.35
	**SD1259**	53	2	IDC	Somatic	Frameshift	c.9097dup	p.(Thr3033Asnfs*11)	No	LumB	7	Yes	70	0.43

Pathogenic MS: pathogenic missense; IDC: invasive ductal carcinoma; ILC: Invasive lobular carcinoma.

Supplementary Table 2: List of tumours with germline alteration and status of LOH.

		Genomic locus LOH [WES]		Variant alternate alellic freq [WES]		Genome wide copy number [sWGS]		
Gene	Sample ID	CN_A_B	Presence of LOH?	Tf-Gf	Allele loss	Copy number analysis	Presence of copy loss/gain	Combined call
PALB2	SD1204	2_2_0	Yes	0.30	Yes	No change	No	Yes
	SD0311	1_1_0	Yes	0.08	No	1 copy loss	Yes	Yes
BRCA1	SD0126	3_3_0	Yes	0.40	Yes	1 copy gain	Yes	Yes
	SD0128	2_2_0	Yes	0.23	Yes	No change	No	Yes
	SD0457	2_2_0	Yes	0.59	Yes	1 copy loss	Yes	Yes
	SD0594	2_2_0	Yes	0.09	No	1 copy loss	Yes	Yes
	SD0901	2_2_0	Yes	0.28	Yes	No change	No	Yes
	SD1496	2_2_0	Yes	0.16	No	1 copy loss	Yes	Yes
	SD0342	2_2_0	Yes	0.20	Yes	1 copy loss	Yes	Yes
BRCA2	SD1051	2_2_0	Yes	-0.18	No	1 copy loss	Yes	Yes
	SD0757	2_2_0	Yes	0.27	Yes	1 copy loss	Yes	Yes
	SD1183	2_2_0	Yes	0.23	Yes	1 copy loss	Yes	Yes
	SD1349	2_2_0	Yes	0.47	Yes	1 copy loss	Yes	Yes
	SD1431	2_2_0	Yes	0.38	Yes	1 copy loss	Yes	Yes
	SD1556	2_2_0	Yes	0.18	No	1 copy loss	Yes	Yes
	SD1240	2_2_0	Yes	0.12	No	1 copy loss	Yes	Yes
PALB2	SD0028	4_2_2	No	0.16	No	1 copy gain	Yes	No
	SD0483	2_1_1	No	0.08	No	No change	No	No
	SD1054	2_1_1	No	0.27	Yes	No change	No	No
	SD0221	2_1_1	No	-0.19	No	No change	No	No
BRCA1	SD0092	2_1_1	No	0.10	No	No change	No	No
	SD1008	2_1_1	No	0.47	Yes	No change	No	No
	SD1177	2_1_1	No	-0.12	No	No change	No	No
BRCA2	SD1245	2_1_1	No	0.02	No	No change	No	No
	SD0163	2_1_1	No	-0.24	No	1 copy loss	Yes	No
	SD0881	2_1_1	No	-0.10	No	No change	No	No
	SD1121	2_1_1	No	-0.10	No	No change	No	No

ASCN analysis as per Sequenza output [B>0: LOH negative; B=0: LOH positive]. Tf: variant alternate allele frequency in tumour; Gf: variant alternate allele frequency in germline. If Tf-Gf>0.20: tumour sample have loss of wildtype allele.

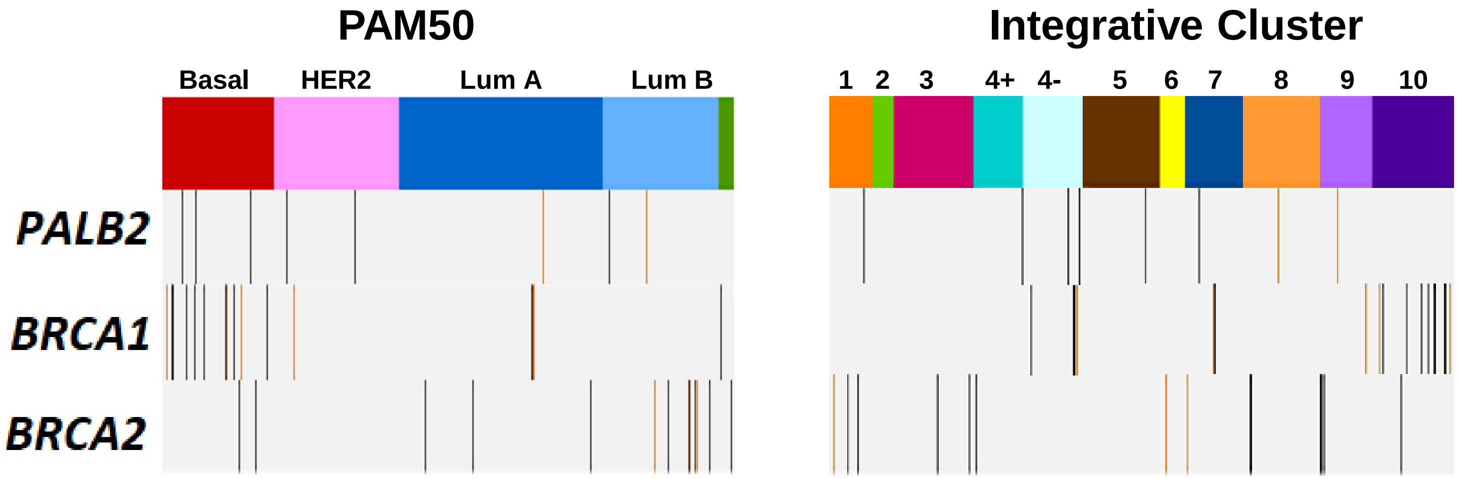

Supplementary Figure 1. Distribution of breast cancer subtype by gene expression data using PAM50 and Integrative Cluster classification. The plot compares the distribution of subtype of tumour with integrated germline and somatic alterations in *PALB2* with *BRCA1*, *BRCA2* and tumours with no alterations that arise from non-carriers. Number of samples included in this analysis (excluded those with no RNA-seq data) – *PALB2:* 8; *BRCA1*: 16; *BRCA2*: 13; Non-carriers: 473; "Black" vertical line represents germline PTV carriers whilst the "orange" line represents somatic PTV carriers. The grey space in between carriers comprised of non-carriers.

a.

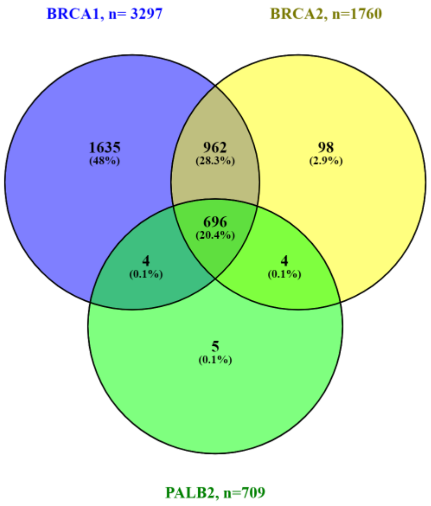

b. **GO functional enrichment analysis**

Source	Category/Term	Name	Bonferroni-adjusted p-value *PALB2* vs NC	*BRCA1* vs NC	*BRCA2* vs NC
GO	Molecular Function / GO:0044822	Poly(A) RNA binding	1.24E-91	7.02E-193	1.24E-138
GO	Molecular Function / GO:0005515	Protein binding	2.14E-58	4.12E-163	1.21E-102
GO	Cellular Component / GO:0016020	Membrane	1.22E-40	2.13E-110	3.98E-91
GO	Cellular Component / GO:0005654	Nucleoplasm	9.85E-39	2.05E-139	1.41E-72
GO	Cellular Component / GO:0005829	Cytosol	2.49E-36	2.71E-110	2.76E-78
GO	Cellular Component / GO:0070062	Extracellular exosome	5.73E-35	5.56E-75	2.73E-61
GO	Biological Process / GO:0000398	mRNA splicing, via spliceosome	1.71E-29	2.36E-35	2.70E-31
GO	Molecular Function / GO:0003723	RNA binding	8.18E-24	9.37E-40	2.02E-35
GO	Cellular Component / GO:0030529	Intracellular ribonucleoprotein complex	1.04E-23	9.88E-30	6.10E-28
GO	Molecular Function / GO:0098641	Cadherin binding involved in cell-cell adhesion	1.21E-22	2.91E-53	8.23E-35

c. **Pathway enrichment analysis based on KEGG and REACTOME**

Source	Category/ Term	Name	Bonferroni-adjusted p-value *PALB2* vs NC	*BRCA1* vs NC	*BRCA2* vs NC
REACTOME	Metabolism of RNA / R-HSA-72163	Splicing of mRNA - major pathway	4.2E-26	4.0E-37	2.5E-26
KEGG	Transcription / hsa03040	Spliceosome	4.6E-15	4.1E-21	4.6E-15
KEGG	Folding, sorting and degradation / hsa04141	Protein processing in endoplasmic reticulum	5.5E-13	1.2E-24	2.5E-18
REACTOME	Metabolism of RNA / R-HSA-72203	Processing of capped intron-containing pre-mRNA	1.1E-11	1.2E-09	2.6E-06
KEGG	Translation / hsa03013	RNA transport	1.9E-06	9.4E-13	2.9E-11
REACTOME	Metabolism of protein / R-HSA-6807878	COPI-mediated anterograde transport	3.1E-05	3.9E-05	2.5E-07
REACTOME	Metabolism of RNA / R-HSA-72187	mRNA 3'-end processing	1.9E-04	6.6E-07	4.9E-05
REACTOME	Metabolism of RNA / R-HSA-159236	Transport of Mature mRNA derived from an Intron-Containing Transcript	2.0E-04	1.9E-11	1.6E-06
REACTOME	Signal transduction / R-HSA-5625740	RHO GTPases activate PKNs	3.0E-04	5.6E-01	1.2E-01
REACTOME	Metabolism of protein / R-HSA-1799339	SRP-dependent cotranslational protein targeting to membrane	4.2E-04	2.7E-24	8.2E-15

GO, Gene Ontology
KEGG, Kyoto Encyclopedia of Genes and Genomes

Supplementary Figure 2. Differentially expressed Genes (DEGs) a. Venn diagram showing top significant DEGs after applying a more stringent cutoff (-log 10 p-value ≥ 50 ; |FC| ≥ 5) which overlap across 3 comparisons [*PALB2* vs Non-carrier; *BRCA1* vs Non-carrier; *BRCA2* vs Non-carrier] b-c. Table showing the biological themes that are over-represented in the top significant DEGs in *PALB2* tumours based on Gene Ontology (GO) enrichment analysis and pathway enrichment analysis.

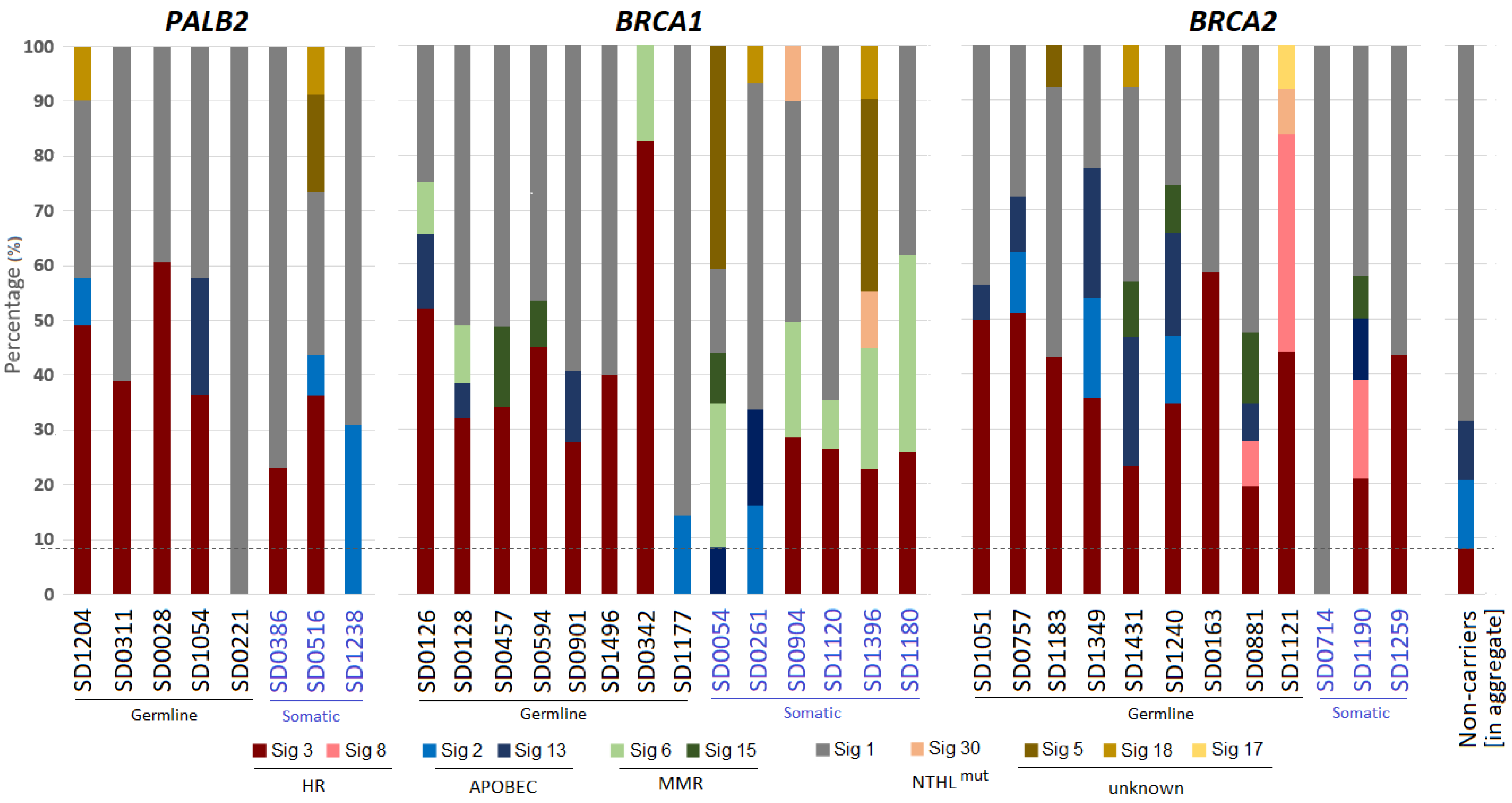

Supplementary Figure 3. The stacked bar plot shows the proportion of major mutational signature for each individual mutation carrier. The horizontal grey dashed line indicated the proportion of mutational signature 3 in tumours that arise from non-carriers as reference for comparison. Number of samples included in this analysis (excluded those with <15 SNVs) – *PALB2*: 8; *BRCA1*: 14; *BRCA2:* 12; Non-carriers (n=483, proportion in aggregate).

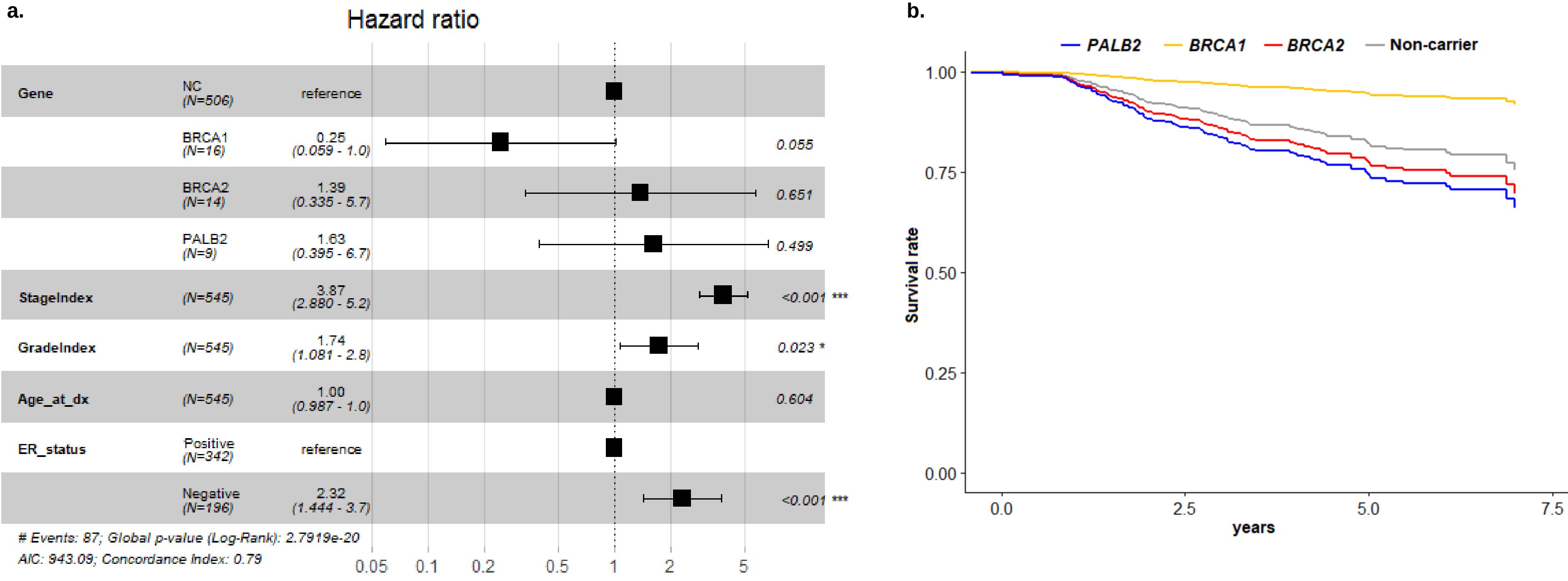

Supplementary Figure 4. Overall survival in patients with germline and somatic alterations in *PALB2*, *BRCA1* and *BRCA2* a. Cox Proportional Hazard model of overall survival. b. Adjusted survival curves were calculated based on Cox model. The analysis included all patients who had events (including those less than 3 years) and all patients who were alive were followed up for at least 3 years. Duration of follow-up is 3.0 – 7.5 years (mean: 4.7 years). Number of events – *PALB2*: 3; *BRCA1*: 3; *BRCA2*: 2; Non-carrier (NC): 85.
