# Supplementary material for: Characterisation of PALB2 tumours through whole-exome and whole-transcriptomic analyses
Source: NPJ Breast Cancer. 2021 Apr 23;7:46. doi: 10.1038/s41523-021-00254-4 (PMC8065101; doi:10.1038/s41523-021-00254-4)
Supplement: Supplementary file 3 — Reporting Summary [file 41523_2021_254_MOESM3_ESM.pdf]

nature research

Corresponding author(s): Soo-Hwang Teo

Last updated by author(s): Nov 26, 2020

Reporting Summary

Nature Research wishes to improve the reproducibility of the work that we publish. This form provides structure for consistency and transparency in reporting. For further information on Nature Research policies, see our Editorial Policies and the Editorial Policy Checklist.

Statistics

For all statistical analyses, confirm that the following items are present in the figure legend, table legend, main text, or Methods section.

n/a	Confirmed	
☐	☒	The exact sample size (*n*) for each experimental group/condition, given as a discrete number and unit of measurement
☐	☒	A statement on whether measurements were taken from distinct samples or whether the same sample was measured repeatedly
☐	☒	The statistical test(s) used AND whether they are one- or two-sided<br>*Only common tests should be described solely by name; describe more complex techniques in the Methods section.*
☐	☒	A description of all covariates tested
☐	☒	A description of any assumptions or corrections, such as tests of normality and adjustment for multiple comparisons
☐	☒	A full description of the statistical parameters including central tendency (e.g. means) or other basic estimates (e.g. regression coefficient) AND variation (e.g. standard deviation) or associated estimates of uncertainty (e.g. confidence intervals)
☐	☒	For null hypothesis testing, the test statistic (e.g. *F*, *t*, *r*) with confidence intervals, effect sizes, degrees of freedom and *P* value noted<br>*Give P values as exact values whenever suitable.*
☒	☐	For Bayesian analysis, information on the choice of priors and Markov chain Monte Carlo settings
☒	☐	For hierarchical and complex designs, identification of the appropriate level for tests and full reporting of outcomes
☒	☐	Estimates of effect sizes (e.g. Cohen's *d*, Pearson's *r*), indicating how they were calculated

Our web collection on statistics for biologists contains articles on many of the points above.

Software and code

Policy information about availability of computer code

Data collection	Standard, open-source tools used in data collection are described in the Methods section.
Data analysis	All computational tools used in data analysis are open-source tools that have been previously published, and are described in the Methods section.

For manuscripts utilizing custom algorithms or software that are central to the research but not yet described in published literature, software must be made available to editors and reviewers. We strongly encourage code deposition in a community repository (e.g. GitHub). See the Nature Research guidelines for submitting code & software for further information.

Data

Policy information about availability of data

All manuscripts must include a data availability statement. This statement should provide the following information, where applicable:

- Accession codes, unique identifiers, or web links for publicly available datasets
- A list of figures that have associated raw data
- A description of any restrictions on data availability

Access to patient sequence data is controlled to protect patient privacy, and thus requires the approval of the Data Access Committee. Requests to access the data should be submitted to genetics@cancerresearch.my.

Field-specific reporting

Please select the one below that is the best fit for your research. If you are not sure, read the appropriate sections before making your selection.

☒ Life sciences ☐ Behavioural & social sciences ☐ Ecological, evolutionary & environmental sciences

For a reference copy of the document with all sections, see nature.com/documents/nr-reporting-summary-flat.pdf

Life sciences study design

All studies must disclose on these points even when the disclosure is negative.

Sample size	The sample size was set in order to detect somatic mutation frequencies of 3% or above as described previously by Pan et al., 2020 Nature Communications. With 560 samples, the sample size also enables general and subtype-specific molecular comparisons of breast cancer to similar genomic studies of breast cancer in other published studies or datasets such as TCGA and METABRIC.
Data exclusions	Patients were excluded from this study for the following criteria: No corresponding germline samples (n=5) and those who withdrew consent (n=12). Tumour samples were further excluded after clinicopathological review if they were found to be from rare histological subtypes and other breast diseases (n=5). Tumour samples with an average tumour content of <30% (n=50) and those with insufficient DNA (n=8) were excluded from the study. After sequencing, samples that did not reach standard sequencing quality metrics were also excluded.
Replication	Where appropriate, all major findings were compared to previously published studies (Li et al. npj Breast Cancer 201, Lee et al. 2018). Some findings could not be compared to those studies due to lack of data or inadequate sample size.
Randomization	Given the nature of the study, randomization was not relevant as comparator groups were based on presence/absence of germline or somatic mutation in PALB2, BRCA1 or BRCA2 and no mutations.
Blinding	Not relevant to the study as comparisons made were made between different groups based on presence/absence of mutation. Additionally, the data analysis team used de-identified patient data and were not involved in patient recruitment or data collection.

Reporting for specific materials, systems and methods

We require information from authors about some types of materials, experimental systems and methods used in many studies. Here, indicate whether each material, system or method listed is relevant to your study. If you are not sure if a list item applies to your research, read the appropriate section before selecting a response.

Materials & experimental systems

n/a	Involved in the study	
☐	☒	Antibodies
☒	☐	Eukaryotic cell lines
☒	☐	Palaeontology and archaeology
☒	☐	Animals and other organisms
☐	☒	Human research participants
☒	☐	Clinical data
☒	☐	Dual use research of concern

Methods

n/a	Involved in the study	
☒	☐	ChIP-seq
☒	☐	Flow cytometry
☒	☐	MRI-based neuroimaging

Antibodies

Antibodies used	Antibodies used were anti-CD3 (clone 2GV6, predilute; Ventana Medical Systems), anti-CD4 (clone SD35, predilute; Ventana Medical Systems), anti-CD8 (clone SD57, predilute; Ventana Medical Systems) and anti-PD-1 (clone SP263, predilute; Ventana Medical Systems).
Validation	All antibodies used are commercially available and validated by Roche Diagnostics for in vitro diagnostic (IVD) use in sections of normal and neoplastic human tissues, as listed on the manufacturer's website.

Human research participants

Policy information about studies involving human research participants

Population characteristics	The study population was female, Malaysian breast cancer patients seeking treatment at the Subang Jaya Medical Centre, a private hospital within the Kuala Lumpur metropolitan area.
Recruitment	Patients were recruited sequentially from a hospital-based cohort. The hospital in point, Subang Jaya Medical Centre, is a private Malaysian hospital within the Kuala Lumpur metropolitan area, where patients tend to be of higher socio-economic

status relative to the general population.

Ethics oversight	The project was reviewed and approved by the Independent Ethics Committee, Ramsay Sime Darby Health Care (reference no: 201208.1), and written informed consent was given by each individual patient.

Note that full information on the approval of the study protocol must also be provided in the manuscript.
